# Phenotype-Specific Differences in Insulin Resistance and Androgenic Profiles in Polycystic Ovary Syndrome: A Prospective Observational Study

**DOI:** 10.3390/jcm15187101

**Published:** 2026-09-13

**Authors:** Karolin Ohanoglu Cetinel, Ahmet Cinar, Can Tercan, Emrah Dagdeviren, Yucel Kaya, Sultan Can, Ismail Alay, Nazime Binnur Comert, Kubra Kurt Bilirer, Busra Deniz Gelir, Engin Oral

**Affiliations:** 1 Department of Obstetrics and Gynecology, Basaksehir Cam and Sakura City Hospital, 34480 Istanbul, Türkiyeamaurosorceress@gmail.com (N.B.C.); 2 Department of Obstetrics and Gynecology, Erzurum City Hospital, 25240 Erzurum, Türkiye; 3 Department of Obstetrics and Gynecology, Perinatology, Basaksehir Cam and Sakura City Hospital, 34480 Istanbul, Türkiye; 4 Obstetrics and Gynecology, Private Practice, 34740 Istanbul, Türkiye; drsultancan@gmail.com; 5 Department of Obstetrics and Gynecology, Medicana Bahcelievler Hospital, 34180 Istanbul, Türkiye; 6 Department of Obstetrics and Gynecology, Tekirdağ Hayrabolu State Hospital, 59400 Tekirdag, Türkiye; 7 Department of Clinical Sciences, Norrland University Hospital, Umeå University, 90185 Umeå, Sweden

**Keywords:** polycystic ovary syndrome, PCOS phenotypes, Rotterdam criteria, insulin resistance, HOMA-IR, hyperandrogenism, free androgen index, metabolic risk

## Abstract

**Background and Objectives:** Polycystic ovary syndrome (PCOS) is a heterogeneous endocrine and metabolic disorder characterized by distinct phenotypic presentations. This study aimed to compare androgenic and metabolic characteristics across Rotterdam-defined PCOS phenotypes, with particular emphasis on insulin resistance. **Materials and Methods:** This prospective observational study included 261 nulliparous women with PCOS who were prospectively enrolled and classified according to the Rotterdam criteria as phenotype A (*n* = 101), B (*n* = 39), C (*n* = 55), or D (*n* = 66). Clinical hyperandrogenism, biochemical androgen parameters, hormonal parameters, lipid profiles, and insulin resistance assessed using the homeostasis model assessment of insulin resistance (HOMA-IR) were compared across phenotypes. Insulin resistance was defined as HOMA-IR ≥ 2.5. Multivariable logistic regression was performed to evaluate the association between PCOS phenotype and insulin resistance after adjustment for age and body mass index (BMI). Results: Significant differences were observed among phenotypes in sex hormone-binding globulin (SHBG), free androgen index (FAI), and Ferriman–Gallwey scores (*p* = 0.005, *p* < 0.001, and *p* < 0.001, respectively). Median HOMA-IR differed significantly among phenotypes (*p* < 0.001) and was lowest in phenotype D. The prevalence of insulin resistance was 58.3%, 56.4%, 49.1%, and 22.6% in phenotypes A, B, C, and D, respectively (*p* < 0.001). After adjustment for age and BMI, phenotypes A (adjusted odds ratio [aOR] 4.56, 95% CI 2.20–9.42), B (aOR 4.54, 95% CI 1.90–10.87), and C (aOR 3.29, 95% CI 1.47–7.38) were associated with higher odds of insulin resistance compared with phenotype D. HDL-C also differed among phenotypes (*p* = 0.006), whereas other conventional lipid parameters, the TG/HDL ratio, and AIP were comparable. **Conclusions:** Rotterdam-defined PCOS phenotypes exhibit distinct androgenic and metabolic profiles. Hyperandrogenic phenotypes, particularly A and B, were associated with greater insulin resistance, whereas phenotype D showed a more favorable metabolic profile. These findings reflect cross-sectional metabolic differences between PCOS phenotypes and should not be interpreted as evidence of future cardiometabolic risk.

## 1. Introduction

Polycystic ovary syndrome (PCOS) is a common endocrine disorder affecting women of reproductive age and is associated with a wide range of reproductive, metabolic, and androgen-related disturbances [1]. The reported prevalence of PCOS varies according to the diagnostic criteria used and the characteristics of the studied population, but estimates suggest that approximately 10–15% of women worldwide are affected [2]. Owing to its considerable clinical and biochemical variability, PCOS is increasingly recognized as a heterogeneous condition rather than a single uniform entity [1,3,4]. Recently, an international consensus process renamed PCOS as polyendocrine metabolic ovarian syndrome (PMOS) to better reflect its endocrine and metabolic features [5]; as the present cohort was diagnosed and classified under the Rotterdam PCOS framework, the term PCOS is retained throughout this manuscript for consistency with the diagnostic criteria and cited literature.

The Rotterdam criteria define PCOS by the presence of at least two of the following features: hyperandrogenism (clinical and/or biochemical), oligo- or anovulation, and polycystic ovarian morphology on ultrasonography [6]. Based on different combinations of these features, four phenotypic presentations have been described: phenotype A (hyperandrogenism, oligo/anovulation, and polycystic ovarian morphology), phenotype B (hyperandrogenism and oligo/anovulation), phenotype C (hyperandrogenism and polycystic ovarian morphology), and phenotype D (oligo/anovulation and polycystic ovarian morphology without evidence of hyperandrogenism) [4]. Previous studies have suggested that these phenotypes differ in terms of clinical presentation, degree of androgen excess, insulin resistance, and cardiometabolic risk [7,8].

Hyperandrogenism is a key feature of PCOS and contributes substantially to its reproductive and clinical manifestations. Clinical hyperandrogenism is commonly assessed using the Ferriman–Gallwey scoring system [9], whereas biochemical hyperandrogenism is evaluated using measurements of total testosterone, sex hormone-binding globulin (SHBG), and derived indices such as the free androgen index (FAI). Because circulating testosterone is largely bound to SHBG, FAI is considered a useful surrogate marker of androgen bioavailability and may better reflect androgen excess than total testosterone alone in some women with PCOS [10]. However, increasing evidence suggests that PCOS is also characterized by considerable metabolic heterogeneity. Insulin resistance, dyslipidemia, and cardiometabolic risk factors are frequently observed in women with PCOS, although their prevalence and severity appear to vary across different phenotypic presentations [7,8,11,12]. Several studies have suggested that the classic hyperandrogenic phenotypes may exhibit a less favorable metabolic profile than non-hyperandrogenic phenotypes, but findings have been inconsistent across populations and study designs [7,8].

Phenotype-based classification has therefore gained increasing importance not only for characterizing androgen excess but also for identifying women at greater metabolic risk. While clinical manifestations of hyperandrogenism have been extensively investigated, comparative data integrating androgen-related parameters with markers of insulin resistance and atherogenic lipid profiles across Rotterdam-defined phenotypes remain relatively limited and sometimes inconsistent [8,11]. A better understanding of these phenotype-specific differences may improve risk stratification and facilitate more individualized clinical management of women with PCOS.

Accordingly, the present study aimed to compare clinical and biochemical markers of hyperandrogenism, metabolic characteristics, and insulin resistance across Rotterdam-defined PCOS phenotypes. Specifically, we evaluated Ferriman–Gallwey scores, free androgen index, lipid parameters, atherogenic lipid indices, and HOMA-IR values to determine whether metabolic risk differs according to phenotypic presentation. A phenotype-based evaluation may contribute to a more comprehensive understanding of the endocrine and metabolic heterogeneity of PCOS.

## 2. Materials and Methods

### 2.1. Study Design and Participants

This prospective observational study with a cross-sectional analysis was conducted at Başakşehir Çam and Sakura City Hospital, a tertiary referral center for gynecology and reproductive endocrinology, between August 2021 and November 2025. Following ethics committee approval, eligible women presenting to the outpatient clinic were prospectively enrolled after providing written informed consent. At enrollment, participants underwent clinical examination, including modified Ferriman–Gallwey scoring and anthropometric assessment, and relevant clinical information was recorded prospectively. Blood samples for hormonal and metabolic assessments were subsequently obtained as part of the study protocol. A total of 261 nulliparous women fulfilling the Rotterdam diagnostic criteria and with complete phenotype classification were included in the present cross-sectional analysis. An a priori sample size calculation was performed using G*Power version 3.1.9.7 (Heinrich Heine University Düsseldorf, Düsseldorf, Germany). Based on a one-way ANOVA comparing FAI across the four phenotype groups, with an assumed effect size of 0.30, an alpha level of 0.05, and 80% power, a minimum total sample size of 190 participants was required. A total of 261 eligible women were ultimately included during the predefined study period.

PCOS was diagnosed according to the Rotterdam criteria, in line with the 2023 International Evidence-Based.

Guideline for the Assessment and Management of PCOS [13], based on the presence of at least two of the following three features:

(1) clinical and/or biochemical hyperandrogenism,
(2) oligo/anovulation, and
(3) polycystic ovarian morphology on transvaginal ultrasonography.

Women with known or previously diagnosed congenital adrenal hyperplasia, androgen-secreting tumors, Cushing syndrome, thyroid dysfunction, or hyperprolactinemia were excluded. Women receiving metformin or combined oral contraceptives at the time of enrollment were also excluded to minimize the potential effects of these treatments on metabolic and androgen-related parameters. Patients receiving insulin therapy for pre-existing type 1 or type 2 diabetes mellitus were not excluded. All participants underwent routine hormonal evaluation, including thyroid function tests and prolactin measurements, to exclude secondary causes of hyperandrogenism. All clinical and laboratory assessments included in the present analysis were performed at baseline, before initiation of any treatment based on the study evaluation. Treatment response and subsequent follow-up were not included in the present analysis.

The study protocol was approved by the Local Ethics Committee of Basaksehir Cam and Sakura City Hospital (approval number: 2021.08.169). All procedures were conducted in accordance with the Declaration of Helsinki. Written informed consent was obtained from all participants prior to enrollment.

Participants were classified into four PCOS phenotypes according to the Rotterdam criteria:

- Phenotype A: hyperandrogenism + oligo/anovulation + polycystic ovarian morphology
- Phenotype B: hyperandrogenism + oligo/anovulation
- Phenotype C: hyperandrogenism + polycystic ovarian morphology
- Phenotype D: oligo/anovulation + polycystic ovarian morphology without hyperandrogenism

Clinical hyperandrogenism was assessed based on the presence of hirsutism, with the modified Ferriman–Gallwey (mFG) scoring system used to quantify the degree of terminal hair growth [9]. An mFG score ≥ 8 was considered indicative of clinical hyperandrogenism. Participants were classified into the four PCOS phenotypes according to the Rotterdam criteria based on the clinical and diagnostic information recorded at enrollment. Biochemical androgen parameters, including total testosterone, SHBG, and FAI, were additionally evaluated as study variables.

### 2.2. Clinical and Laboratory Assessment

Demographic and clinical data, including age, body mass index (BMI), smoking status, alcohol consumption, medical history, medication use, and infertility status, were recorded. BMI was calculated as weight (kg) divided by height squared (m^2^).

Venous blood samples were obtained after an overnight fast of at least 8 h during the early follicular phase of the menstrual cycle (cycle days 2–3). In women with amenorrhea, samples were collected at a random time.

All hormonal analyses were performed in the same institutional biochemistry laboratory using automated electrochemiluminescence immunoassay (ECLIA) methods (Roche Diagnostics, Mannheim, Germany). Serum anti-Müllerian hormone (AMH) and total testosterone levels were measured using Elecsys assays on a cobas e 402/cobas e 801 analyzers. SHBG, DHEA-S, prolactin, TSH, LH, FSH, estradiol, and 17-OH progesterone were analyzed using standardized automated immunoassays according to the manufacturer’s instructions. Internal quality controls and calibration procedures were performed regularly in accordance with laboratory standards. Total testosterone levels > 0.82 ng/mL were considered elevated according to the upper limit of the institutional laboratory reference range.

The free androgen index (FAI) was calculated using total testosterone and SHBG concentrations expressed in molar units. Because total testosterone was reported in ng/mL, values were converted to nmol/L using a conversion factor of 3.467 before calculation. FAI was calculated as follows:

FAI = [total testosterone (ng/mL) × 3.467 × 100]/SHBG (nmol/L).

FAI was analyzed as a continuous measure of androgen bioavailability; no predefined FAI cutoff was used for phenotype classification.

In a subset of participants, a standard 75-g oral glucose tolerance test (OGTT) was performed after overnight fasting in accordance with American Diabetes Association (ADA) recommendations [14]. Plasma glucose levels were measured at fasting (0 min), 60 min, and 120 min. However, OGTT-derived measures were not included in the primary analyses because of incomplete data.

Polycystic ovarian morphology (PCOM) was assessed by transvaginal ultrasonography performed by experienced gynecologists. PCOM was defined according to the Rotterdam criteria as the presence of ≥12 follicles measuring 2–9 mm in diameter and/or an ovarian volume > 10 mL in at least one ovary [6]. Ultrasonographic examinations were performed using a Hitachi ARIETTA 65 ultrasound system (Hitachi, Tokyo, Japan).

### 2.3. Metabolic Assessment

Fasting glucose, serum insulin, triglyceride, total cholesterol, low-density lipoprotein cholesterol (LDL-C), and high-density lipoprotein cholesterol (HDL-C) levels were obtained from routine biochemical analyses performed at the institutional laboratory. Serum insulin concentrations were measured using the Elecsys Insulin assay based on an electrochemiluminescence immunoassay (ECLIA) sandwich principle on a Roche cobas e801 analyzer (Roche Diagnostics, Mannheim, Germany). Insulin resistance was estimated using the homeostasis model assessment of insulin resistance (HOMA-IR), calculated as:

HOMA-IR = [fasting glucose (mg/dL) × fasting insulin (µIU/mL)]/405.

For categorical analyses, insulin resistance was defined as HOMA-IR ≥ 2.5, used as an operational cutoff based on previous data in a Turkish PCOS population [15].

The triglyceride-to-HDL cholesterol ratio (TG/HDL ratio) was calculated by dividing fasting triglyceride concentrations by HDL-C concentrations, both expressed in mg/dL. For calculation of the atherogenic index of plasma (AIP), triglyceride and HDL-C concentrations were converted from mg/dL to mmol/L (triglycerides: mg/dL ÷ 88.57; HDL-C: mg/dL ÷ 38.67). AIP was then calculated as log_10_ [triglycerides (mmol/L)/HDL-C (mmol/L)].

### 2.4. Statistical Analysis

Statistical analyses were performed using SPSS version 29.0 (IBM Corp., Armonk, NY, USA). Normality of data distribution was assessed using the Kolmogorov–Smirnov test. Normally distributed variables were expressed as mean ± standard deviation, while non-normally distributed variables were presented as median (25th–75th percentile). Categorical variables were expressed as numbers and percentages.

Comparisons among PCOS phenotypes were conducted using one-way ANOVA or the Kruskal–Wallis test for continuous variables and the chi-square or Fisher’s exact test for categorical variables, as appropriate. For categorical variables with small expected cell counts in contingency tables larger than 2 × 2, the Fisher–Freeman–Halton exact test was used. For variables demonstrating significant overall differences in Kruskal–Wallis analyses, Bonferroni-adjusted pairwise comparisons were performed to identify inter-phenotype differences. The prevalence of insulin resistance (HOMA-IR ≥ 2.5) was compared among phenotypes using the chi-square test. To assess whether the association between phenotype and insulin resistance was independent of age and BMI, a multivariable logistic regression model was performed with insulin resistance (HOMA-IR ≥ 2.5) as the dependent variable, PCOS phenotype (reference: phenotype D), age, and BMI as covariates; results are presented as adjusted odds ratios (aORs) with 95% confidence intervals (CIs).

A *p* value < 0.05 was considered statistically significant. Given the exploratory nature of phenotype-based comparisons, adjustments for multiple testing were limited to post hoc pairwise analyses.

## 3. Results

The study included 261 women with PCOS who were classified into four phenotypes (A–D) according to the Rotterdam criteria: phenotype A (*n* = 101), phenotype B (*n* = 39), phenotype C (*n* = 55), and phenotype D (*n* = 66). Due to missing data in some variables, analyses were performed using available-case denominators.

Age and BMI did not differ significantly across PCOS phenotypes A–D (*p* = 0.274 and *p* = 0.067, respectively). Marital status, smoking, and alcohol use were similarly distributed across phenotypes (*p* = 0.935, *p* = 0.414, and *p* = 0.353, respectively). The prevalence of diabetes mellitus, hypertension, and infertility was low and did not differ significantly among phenotypes (*p* = 0.852, *p* = 0.358, and *p* = 0.603, respectively) (Table 1).

Median levels of FSH, LH, estradiol, prolactin, TSH, and AMH were comparable across PCOS phenotypes A–D, with no statistically significant differences observed for any of these parameters (all *p* > 0.05) (Table 2).

Table 3 summarizes androgen-related biochemical parameters and clinical hyperandrogenism across PCOS phenotypes A–D. Significant differences were observed among phenotypes in SHBG, FAI, and Ferriman–Gallwey (FG) scores (*p* = 0.005, *p* < 0.001, and *p* < 0.001, respectively), whereas total testosterone, 17-OH progesterone, and DHEA-S levels did not differ significantly among groups (*p* = 0.071, *p* = 0.774, and *p* = 0.214, respectively). Median FAI values were 4.66 (2.77–7.37), 2.50 (1.68–4.20), 3.51 (1.72–5.48), and 2.87 (1.63–5.06) in phenotypes A, B, C, and D, respectively. Post-hoc comparisons showed significantly higher FAI values in phenotype A than in phenotypes B and D. SHBG concentrations were significantly lower in phenotype A than in phenotype D. FG scores also differed across phenotypes, with the lowest values observed in phenotype D. These differences in androgen-related measures should be interpreted in the context of the clinical and/or biochemical hyperandrogenism criteria used in Rotterdam phenotype classification.

Exploratory correlation analyses were additionally performed between androgen-related and metabolic parameters. FAI showed weak but statistically significant correlations with several metabolic parameters, including BMI and lipid-related measures, but was not significantly correlated with HOMA-IR. Directly measured free testosterone was not significantly associated with the evaluated metabolic parameters. No significant correlation was observed between AMH and HOMA-IR (Spearman’s ρ = 0.009, *p* = 0.894; *n* = 243). Detailed correlation results are provided in Supplementary Table S1.

Metabolic parameters according to PCOS phenotype are presented in Table 4. Median HOMA-IR values differed significantly among phenotypes (*p* < 0.001), with values of 2.95 (1.88–4.91), 3.00 (1.90–4.20), 2.40 (1.70–3.50), and 1.85 (1.40–2.40) in phenotypes A, B, C, and D, respectively. Post-hoc comparisons showed significantly lower HOMA-IR values in phenotype D than in phenotypes A, B, and C. HDL-C concentrations also differed significantly across phenotypes (*p* = 0.006); phenotype C had significantly higher HDL-C levels than phenotypes A and D, whereas phenotype B did not differ significantly from the other phenotypes. Fasting glucose, triglyceride, LDL-C, and total cholesterol levels were comparable among groups (all *p* > 0.05). The TG/HDL ratio did not differ significantly among phenotypes (*p* = 0.072). After calculation using triglyceride and HDL-C concentrations expressed in mmol/L, median AIP values were −0.10 (−0.27–0.09), −0.19 (−0.33–0.05), −0.19 (−0.35–−0.08), and −0.13 (−0.29–0.06) in phenotypes A, B, C, and D, respectively, with no significant overall difference (*p* = 0.072).

Post-hoc pairwise comparisons of HOMA-IR are summarized in Table 5. Phenotype D demonstrated significantly lower HOMA-IR values than phenotype A (*p* < 0.001), phenotype B (*p* = 0.003), and phenotype C (*p* = 0.047). No significant differences were observed among the hyperandrogenic phenotypes A, B, and C. Thus, within this cohort, phenotype D was characterized by lower HOMA-IR values than the three hyperandrogenic phenotypes.

The prevalence of insulin resistance, defined as HOMA-IR ≥ 2.5, differed significantly among PCOS phenotypes (*p* < 0.001). Insulin resistance was observed in 58.3% of phenotype A, 56.4% of phenotype B, 49.1% of phenotype C, and 22.6% of phenotype D. Consistent with the continuous HOMA-IR analysis, phenotype D had the lowest observed prevalence of HOMA-IR-defined insulin resistance in this cohort (Table 6).

To determine whether the association between PCOS phenotype and insulin resistance was independent of age and body mass index, a multivariable logistic regression model was constructed with insulin resistance (HOMA-IR ≥ 2.5) as the dependent variable, phenotype D as the reference category, and age and BMI as covariates (*n* = 250). Phenotypes A, B, and C were each associated with significantly higher odds of insulin resistance compared with phenotype D, independent of age and BMI (overall likelihood ratio test for phenotype, 3 degrees of freedom, *p* < 0.001) (Table 7).

## 4. Discussion

In this cohort of women with PCOS, both androgenic and metabolic characteristics differed across Rotterdam-defined phenotypes. These findings further support the concept that PCOS represents a heterogeneous syndrome comprising distinct phenotypic presentations with varying endocrine and metabolic profiles [4]. Phenotype A showed higher androgen-related measures, whereas phenotype D showed the lowest HOMA-IR values and the lowest prevalence of insulin resistance. HDL cholesterol levels differed significantly across phenotypes, with the highest concentrations observed in phenotype C.

The differences observed in Ferriman–Gallwey scores, SHBG, and FAI are consistent with the established concept that the classic phenotypes represent the more severe end of the PCOS spectrum [16,17]. Hyperandrogenism is a defining feature of PCOS and contributes to many of its reproductive and clinical manifestations [11,17]. As expected from the Rotterdam phenotype definitions, phenotype A showed higher androgen-related measures, whereas phenotype D showed the lowest androgen-related measures. Similar findings have been reported by Carmina and Lobo [16] and more recently by Ma et al. [18], who observed that phenotype A was associated with more pronounced endocrine abnormalities than other phenotypes.

FAI differed significantly across phenotypes, whereas total testosterone alone did not. While total testosterone did not differ significantly among phenotypes, marked differences were observed in FAI, highlighting the potential value of incorporating SHBG into the assessment of androgen status. This observation is biologically plausible, as SHBG concentrations influence the biologically active fraction of circulating testosterone [10,19]. Lower SHBG levels in the classic phenotypes likely contributed to the higher FAI values observed in our cohort. Recent studies have increasingly emphasized the limitations of total testosterone as a solitary marker of biochemical hyperandrogenism and highlighted the additional value of indices that incorporate SHBG concentrations [20].

The most striking metabolic finding was the difference in insulin resistance across phenotypes. Median HOMA-IR values were highest in phenotypes A and B and lowest in phenotype D. A similar pattern was observed when insulin resistance was analyzed categorically, with only 22.6% of women in phenotype D meeting the criterion for insulin resistance compared with approximately half of those in the hyperandrogenic phenotypes. These findings are in line with previous reports indicating that metabolic dysfunction is more pronounced in hyperandrogenic forms of PCOS [7,8,18]. Importantly, in multivariable logistic regression adjusting for age and BMI, phenotypes A, B, and C remained independently associated with significantly higher odds of insulin resistance relative to phenotype D (Table 7), indicating that this association is not simply attributable to differences in adiposity between phenotypes.

The relationship between androgen excess and insulin resistance has been extensively discussed in the PCOS literature. Hyperinsulinemia stimulates ovarian androgen production and suppresses hepatic SHBG synthesis, thereby increasing androgen bioavailability. At the same time, androgen excess may contribute to worsening insulin sensitivity through its effects on adipose tissue function and metabolic regulation [7,17]. This reciprocal interaction may explain why the phenotypes characterized by greater androgen excess also demonstrated the highest HOMA-IR values in our study.

Our findings are broadly consistent with those of Wen et al. [8], who reported insulin resistance in 66.7% of women with the OD-HA phenotype and 60.8% of those with the HA-PCOM phenotype, compared with 49.1% in the non-hyperandrogenic OD-PCOM group. In their adjusted analyses, testosterone > 1.67 nmol/L (OR 2.01, 95% CI 1.24–3.45) and an F–G score > 3 (OR 2.41, 95% CI 1.31–4.29) were associated with insulin resistance. Although direct numerical comparison is limited by differences in phenotype classification and the HOMA-IR threshold used, these findings similarly suggest an association between hyperandrogenic features and insulin resistance. Similar observations were reported by Ma et al. [18], who found greater metabolic impairment in the hyperandrogenic phenotypes, particularly phenotypes A and B, compared with phenotype D. The agreement between these studies and our findings supports the concept that metabolic risk is not equally distributed across Rotterdam phenotypes. Recent perspectives increasingly emphasize the need to move beyond reproductive manifestations and incorporate phenotype-specific metabolic assessment into routine PCOS management [11,13,21].

HDL cholesterol concentrations also differed significantly among phenotypes, with the highest levels observed in phenotype C. In contrast, triglyceride, LDL cholesterol, total cholesterol, TG/HDL ratio, and AIP did not differ significantly among phenotypes [11,18]. The absence of statistical significance for some lipid-derived indices may be related to the relatively young age of our cohort and the low prevalence of overt metabolic disease. The higher HDL-C levels observed in phenotype C, together with the absence of significant differences in other lipid parameters, suggest that metabolic differences across phenotypes do not follow a uniform pattern and should therefore be interpreted cautiously.

Most hormonal parameters, including FSH, LH, estradiol, prolactin, TSH, DHEA-S, and AMH, were comparable across phenotypes. Similar findings have been reported in previous phenotype-based studies [8,18], suggesting that androgen-related markers may better discriminate between phenotypes than ovarian reserve markers such as AMH. These observations indicate that phenotypic differences in PCOS may be driven primarily by variations in androgen excess and metabolic dysfunction rather than by broad alterations in reproductive hormone profiles.

## 5. Strengths and Limitations

The inclusion of all four Rotterdam phenotypes, the simultaneous assessment of clinical hyperandrogenism, biochemical androgen markers, insulin resistance, and lipid parameters, and the use of multivariable adjustment for age and BMI to confirm the independence of the phenotype-insulin resistance association represent important strengths of this study. In addition, all laboratory analyses were performed in a single tertiary referral center using standardized methods, reducing potential inter-laboratory variability.

Several limitations should be considered when interpreting our findings. First, the cross-sectional design does not allow conclusions regarding causality between hyperandrogenism and metabolic dysfunction. Second, the study population consisted exclusively of nulliparous women from a single center, which may limit the generalizability of the results. Third, the relatively small size of some phenotype groups may have limited statistical power for detecting modest between-group differences. Fourth, although a standard 75-g oral glucose tolerance test (OGTT) was available for a subset of participants, incomplete data and the limited number of examinations within individual phenotypes precluded robust inter-phenotype comparisons. The relatively low availability of OGTT data may partly reflect patient reluctance toward glucose loading tests and intolerance to the procedure in some individuals. Consequently, OGTT-derived measures of glucose metabolism were not included in the primary analyses. In addition, anthropometric measures reflecting central adiposity, such as waist circumference and waist-to-hip ratio, were not consistently available. Given the known relationship between abdominal adiposity and insulin resistance, inclusion of these parameters might have provided additional insight into the metabolic differences among phenotypes. The study protocol was established in 2021, before publication of the 2023 International Evidence-Based Guideline for PCOS. As calculated free testosterone was not included in the original study protocol, a direct comparison between calculated free testosterone and FAI was not possible. FAI was therefore evaluated as a derived marker of androgen bioavailability, and this limitation should be considered when interpreting the androgen-related findings. In women with amenorrhea, hormonal sampling was performed at a random time rather than during a standardized cycle phase, which may have introduced variability in gonadotropin and estradiol measurements. Finally, more direct methods for assessing insulin sensitivity were not available, and longitudinal follow-up data were lacking.

## 6. Conclusions

Rotterdam-defined PCOS phenotypes exhibit distinct androgenic and metabolic characteristics. Hyperandrogenic phenotypes, particularly A and B, were associated with greater insulin resistance and a higher prevalence of insulin resistance, whereas phenotype D displayed a more favorable metabolic profile. Given the cross-sectional design, these findings reflect metabolic differences between phenotypes and should not be interpreted as evidence of future cardiometabolic risk.

## Figures and Tables

**Table 1:** Demographic and general clinical characteristics according to PCOS phenotypes.

Variable	Phenotype A (*n* = 101)	Phenotype B (*n* = 39)	Phenotype C (*n* = 55)	Phenotype D (*n* = 66)	*p* Value
Age (years)	23.48 ± 4.83	25.00 ± 5.05	24.76 ± 5.25	24.27 ± 4.81	0.274
BMI (kg/m^2^)	27.20 (22.60–30.10)	24.20 (22.00–28.40)	24.80 (22.49–27.38)	25.40 (22.00–27.57)	0.067
Marital status, married	13/50 (26.0)	3/10 (30.0)	7/27 (25.9)	8/25 (32.0)	0.935
Smoking, current	3/50 (6.0)	2/10 (20.0)	3/27 (11.1)	2/25 (8.0)	0.414
Alcohol use, yes	2/50 (4.0)	1/10 (10.0)	2/27 (7.4)	0/25 (0.0)	0.353
Diabetes mellitus, yes	2/50 (4.0)	0/10 (0.0)	1/27 (3.7)	0/25 (0.0)	0.852
Hypertension, yes	3/101 (3.0)	0/39 (0.0)	2/55 (3.6)	0/66 (0.0)	0.358
Infertility, yes	4/48 (8.3)	0/9 (0.0)	3/23 (13.0)	1/25 (4.0)	0.603

Notes: Data are presented as mean ± SD for normally distributed variables or median (25th–75th percentile) for non-normally distributed variables. Comparisons of continuous variables were performed using one-way ANOVA or the Kruskal–Wallis test, as appropriate. Categorical variables are presented as *n*/N (%), where *n* represents the number of participants with the characteristic and N represents the number of participants with available data. Categorical variables were compared using the Fisher–Freeman–Halton exact test. A *p*-value < 0.05 was considered statistically significant. Comparisons for variables with substantial missing data should be interpreted as descriptive.

**Table 2:** Hormonal and biochemical parameters according to PCOS phenotypes.

Variable	Phenotype A (*n* = 101)	Phenotype B (*n* = 39)	Phenotype C (*n* = 55)	Phenotype D (*n* = 66)	*p* Value
FSH (IU/L)	5.61 (4.67–6.64) (*n* = 99)	5.91 (5.22–6.83) (*n* = 39)	5.78 (4.76–6.39) (*n* = 53)	5.57 (4.42–6.40) (*n* = 66)	0.246
LH (IU/L)	8.76 (5.85–12.45) (*n* = 98)	6.67 (5.12–11.50) (*n* = 39)	7.80 (4.77–11.62) (*n* = 52)	8.38 (5.79–12.30) (*n* = 66)	0.492
Estradiol (pg/mL)	40.00 (29.25–51.20) (*n* = 99)	41.70 (32.10–58.60) (*n* = 39)	34.40 (28.80–47.20) (*n* = 53)	38.55 (28.02–51.95) (*n* = 66)	0.154
Prolactin (ng/mL)	13.20 (9.71–20.60) (*n* = 100)	14.10 (11.35–17.70) (*n* = 39)	16.50 (11.00–21.45) (*n* = 55)	14.80 (12.20–19.15) (*n* = 66)	0.588
TSH (mIU/L)	1.96 (1.34–2.66) (*n* = 100)	2.07 (1.39–2.69) (*n* = 38)	2.24 (1.50–2.96) (*n* = 55)	1.83 (1.31–2.24) (*n* = 66)	0.144
AMH (ng/mL)	5.52 (3.58–7.53) (*n* = 96)	4.57 (2.50–7.42) (*n* = 39)	4.96 (3.70–7.04) (*n* = 54)	5.04 (3.47–7.20) (*n* = 65)	0.647

FSH, follicle-stimulating hormone; LH, luteinizing hormone; TSH, thyroid-stimulating hormone; AMH, anti-Müllerian hormone. Data are presented as median (25–75th percentile). Comparisons among PCOS phenotypes were performed using the Kruskal–Wallis test. PCOS phenotypes were defined according to the Rotterdam criteria.

**Table 3:** Androgen-related biochemical parameters and clinical hyperandrogenism according to PCOS phenotype.

Variable	Phenotype A (*n* = 101)	Phenotype B (*n* = 39)	Phenotype C (*n* = 55)	Phenotype D (*n* = 66)	*p* Value
SHBG (nmol/L)	36.80 (27.00–47.20) ^a^	44.05 (30.98–56.00) ^ab^	45.80 (38.60–57.53) ^ab^	46.20 (40.65–60.15) ^b^	0.005
Total testosterone (ng/mL)	0.472 (0.304–0.610)	0.354 (0.248–0.450)	0.396 (0.283–0.580)	0.382 (0.311–0.527)	0.071
Free androgen index (FAI)	4.66 (2.77–7.37) ^a^	2.50 (1.68–4.20) ^b^	3.51 (1.72–5.48) ^ab^	2.87 (1.63–5.06) ^b^	<0.001
17-OH progesterone (ng/mL)	0.48 (0.27–0.62)	0.48 (0.28–0.86)	0.46 (0.24–0.55)	0.48 (0.30–0.70)	0.774
DHEA-S (µg/dL)	285.00 (202.75–373.00)	222.00 (165.00–291.50)	269.00 (196.50–412.00)	261.50 (196.00–368.75)	0.214
Ferriman–Gallwey score	22.00 (17.00–24.00) ^a^	17.00 (14.00–23.00) ^ab^	15.00 (13.00–18.50) ^b^	4.00 (2.00–5.00) ^c^	<0.001

Abbreviations: SHBG, sex hormone-binding globulin; FAI, free androgen index; DHEA-S, dehydroepiandrosterone sulfate; 17-OH progesterone, 17-hydroxyprogesterone. Notes: Data are presented as median (25th–75th percentile). Comparisons among PCOS phenotypes were performed using the Kruskal–Wallis test. For variables with a significant overall difference, Bonferroni-adjusted pairwise comparisons were performed. Different superscript letters indicate statistically significant pairwise differences, whereas groups sharing at least one superscript letter did not differ significantly. For SHBG, phenotype A differed significantly from phenotype D. For FAI, phenotype A differed significantly from phenotypes B and D. For the Ferriman–Gallwey score, phenotype A differed significantly from phenotypes C and D, while phenotypes B and C differed significantly from phenotype D. A *p* value < 0.05 was considered statistically significant.

**Table 4:** Metabolic characteristics according to PCOS phenotypes.

Variable	Phenotype A (*n* = 101)	Phenotype B (*n* = 39)	Phenotype C (*n* = 55)	Phenotype D (*n* = 66)	*p* Value
Fasting glucose (mg/dL)	86.00 (78.00–94.00)	90.50 (81.50–97.50)	87.00 (81.00–92.00)	90.00 (76.00–94.00)	0.918
Triglycerides (mg/dL)	98.00 (74.00–132.00)	91.00 (62.00–136.00)	84.00 (61.50–108.00)	86.50 (61.50–125.00)	0.350
HDL-C (mg/dL)	50.00 (44.00–61.00) ^a^	55.00 (48.00–63.50) ^ab^	62.00 (48.50–71.00) ^b^	49.00 (44.00–59.50) ^a^	0.006
LDL-C (mg/dL)	100.50 (75.00–119.00)	94.00 (81.50–119.00)	97.00 (71.50–119.00)	92.00 (75.00–113.00)	0.748
Total cholesterol (mg/dL)	171.00 (149.00–198.00)	173.00 (147.00–200.00)	169.00 (152.50–192.50)	158.50 (141.25–194.50)	0.559
TG/HDL ratio	1.84 (1.22–2.80)	1.48 (1.07–2.55)	1.49 (1.03–1.91)	1.68 (1.19–2.62)	0.072
AIP	−0.10 (−0.27–0.09)	−0.19 (−0.33–0.05)	−0.19 (−0.35–−0.08)	−0.13 (−0.29–0.06)	0.072
HOMA-IR	2.95 (1.88–4.91) ^a^ (*n* = 96)	3.00 (1.90–4.20) ^a^ (*n* = 39)	2.40 (1.70–3.50) ^a^ (*n* = 53)	1.85 (1.40–2.40) ^b^ (*n* = 62)	<0.001

Abbreviations: HDL-C, high-density lipoprotein cholesterol; LDL-C, low-density lipoprotein cholesterol; TG, triglyceride; AIP, atherogenic index of plasma; HOMA-IR, homeostasis model assessment of insulin resistance. Notes: Data are presented as median (25th–75th percentile). Comparisons among PCOS phenotypes were performed using the Kruskal–Wallis test. For variables with a significant overall difference, Bonferroni-adjusted pairwise comparisons were performed. Different superscript letters indicate statistically significant pairwise differences, whereas groups sharing at least one superscript letter did not differ significantly. For HDL-C, phenotype C had significantly higher levels than phenotypes A and D, while phenotype B did not differ significantly from the other phenotypes. For HOMA-IR, phenotype D had significantly lower values than phenotypes A, B, and C. A *p* value < 0.05 was considered statistically significant.

**Table 5:** Bonferroni-adjusted pairwise comparisons of HOMA-IR.

Comparison	Adjusted *p* Value
Phenotype A vs. B	>0.999
Phenotype A vs. C	0.425
Phenotype A vs. D	<0.001
Phenotype B vs. C	0.894
Phenotype B vs. D	0.003
Phenotype C vs. D	0.047

Notes: HOMA-IR, homeostasis model assessment of insulin resistance. Following a significant overall Kruskal–Wallis test, Bonferroni-adjusted pairwise comparisons were performed. The overall Kruskal–Wallis test was significant (*p* < 0.001). Statistical significance was set at *p* < 0.05.

**Table 6:** Prevalence of insulin resistance (HOMA-IR ≥ 2.5) according to PCOS phenotype.

PCOS Phenotype	HOMA-IR ≥ 2.5 *n*/N (%)
A	56/96 (58.3%)
B	22/39 (56.4%)
C	26/53 (49.1%)
D	14/62 (22.6%)
*p* value	<0.001

Notes: HOMA-IR, homeostasis model assessment of insulin resistance. Insulin resistance was defined as HOMA-IR ≥ 2.5. Comparisons among phenotypes were performed using the chi-square test. Statistical significance was set at *p* < 0.05.

**Table 7:** Multivariable logistic regression for insulin resistance (HOMA-IR ≥ 2.5), adjusted for age and BMI.

Variable	Adjusted OR	95% CI	*p* Value
Phenotype A (vs. D)	4.56	2.20–9.42	<0.001
Phenotype B (vs. D)	4.54	1.90–10.87	<0.001
Phenotype C (vs. D)	3.29	1.47–7.38	0.004
Age (per year)	0.97	0.92–1.02	0.283
BMI (per kg/m^2^)	1.02	0.97–1.07	0.485

Notes: OR, odds ratio; CI, confidence interval; BMI, body mass index; HOMA-IR, homeostasis model assessment of insulin resistance. Reference category: phenotype D. The model included 250 women with complete data for phenotype, age, BMI, and HOMA-IR. Statistical significance was set at *p* < 0.05.

## Data Availability

The dataset is available from the corresponding author upon reasonable request.

## Supplementary Materials

The following supporting information can be downloaded at https://www.mdpi.com/article/10.3390/jcm15187101/s1, Table S1: Correlations between androgen-related parameters and metabolic parameters in women with PCOS.
