# Supplementary material for: Phenotype-Specific Differences in Insulin Resistance and Androgenic Profiles in Polycystic Ovary Syndrome: A Prospective Observational Study
Source: J Clin Med. 2026 Sep 13;15(18):7101. doi: 10.3390/jcm15187101 (PMC13607900; doi:10.3390/jcm15187101)
Supplement: Supplementary file 1 [file jcm-15-07101-s001.zip › jcm-4538424-supplementary.pdf]

Supplementary Table S1. Correlations between androgen-related parameters and metabolic parameters in women with PCOS

p	FAI ϱ	p	Measured free testosterone ϱ	p	Total testosterone ϱ	Metabolic parameter
0.001	0.204	0.396	−0.053	0.030	0.134	BMI
0.008	0.163	0.432	0.049	0.011	0.157	Triglycerides
0.026	0.138	0.967	0.003	0.021	0.143	Total cholesterol
0.001	0.200	0.617	−0.031	0.005	0.173	LDL-C
<0.001	−0.215	0.796	−0.016	<0.001	−0.210	HDL-C
0.081	0.110	0.725	0.022	0.482	0.045	HOMA-IR
<0.001	0.212	0.431	0.049	<0.001	0.207	AIP

Additional exploratory analysis: AMH was not significantly correlated with HOMA-IR (**Spearman's ϱ = 0.009, p = 0.894; n = 243**).

Note: *Correlations were assessed using Spearman's rank correlation coefficient (ρ). FAI, free androgen index; BMI, body mass index; HDL-C, high-density lipoprotein cholesterol; LDL-C, low-density lipoprotein cholesterol; HOMA-IR, homeostasis model assessment of insulin resistance; AIP, atherogenic index of plasma. Free testosterone refers to directly measured serum free testosterone and not calculated free testosterone (cFT).*
